# Does Timing Matter? Perioperative Outcomes and Quality of Life After Early Versus Delayed Radical Cystectomy Following BCG Therapy

**DOI:** 10.3390/jcm15197486

**Published:** 2026-09-26

**Authors:** Yannic Volz, Verena Ghali, Nikolaos Pyrgidis, Matthias P. Fabritius, Benedikt Ebner, Marie Semmler, Marc Kidess, Raphael Scheuringer, Julian Marcon, Patrick Keller, Gerald Bastian Schulz, Christian Stief, Friedrich Jokisch

**Affiliations:** 1 Department of Urology, LMU University Hospital, Marchioninistraße 15, 81377 Munich, Germany; 2 Department of Radiology, LMU University Hospital, Marchioninistraße 15, 81377 Munich, Germany

**Keywords:** bladder cancer, BCG, cystectomy, intravesical therapy, quality of life

## Abstract

**Objectives:** To determine whether the timing of radical cystectomy (RC), either early versus delayed following BCG therapy, affects perioperative outcomes and health-related quality of life (HRQOL) in patients with very-high-risk non–muscle invasive bladder cancer (NMIBC). While existing data focus on oncological endpoints, the impact of BCG on these outcomes remains unevaluated. **Methods:** We retrospectively analysed a prospectively maintained database of 126 patients undergoing RC for very-high-risk NMIBC at a tertiary referral centre, stratified into early RC (n = 81) and delayed RC (n = 45, following BCG therapy). Perioperative outcomes, HRQOL assessed by EORTC-QLQ-C30 at baseline and 3, 12, and 24 months postoperative, and overall survival (OS) were compared. Longitudinal HRQOL changes were analysed using linear mixed-effects models adjusted for age and Carcinoma in situ (CIS) status, correcting for multiple comparisons. **Results:** Groups were well-balanced at baseline regarding age, sex, BMI, and ECOG performance status. No significant between-group differences were observed in overall complication rates, hospital stay length, or any QLQ-C30 domain at any timepoint, including global health status (GHS), in linear mixed-effects models accounting for repeated measures and multiple comparisons. OS did not differ between groups (*p* = 0.720). **Conclusions:** Prior BCG therapy does not adversely influence perioperative outcomes or HRQOL following RC for very-high-risk NMIBC. These findings support the initial use of BCG in eligible patients, as a trial of intravesical therapy does not appear to compromise surgical safety or postoperative quality of life when cystectomy is ultimately required.

## 1. Introduction

Non-muscle invasive bladder cancer (NMIBC) accounts for around 70% of all urothelial cell cancers at first diagnosis [1]. The current EAU risk stratification includes a ‘very high-risk’ group in addition to the established low-, intermediate-, and high-risk categories, based on the probability of progression to muscle-invasive bladder cancer, incorporating tumour stage, grade, size, number, concomitant CIS, and patient age [2]. The current standard of care in the challenging “very-high-risk” population is intravesical therapy using BCG for three years, while early radical cystectomy (RC) may alternatively be discussed upfront in selected cases [3]. However, both therapies are not devoid of side effects. BCG instillation therapy is associated with a broad spectrum of adverse events: the most frequent local side effects include BCG-induced cystitis, bacterial superinfection, urinary frequency, and macroscopic haematuria, while systemic side effects such as general malaise and fever affect a relevant proportion of patients, and treatment discontinuation due to severe side effects is required in approximately 8% of cases [4].

Furthermore, up to 40% of NMIBC patients ultimately fail intravesical BCG therapy, necessitating radical cystectomy as the standard salvage procedure [5,6,7]. Radical cystectomy itself is the most complex operation in urology. It carries a 30-day mortality of approximately 2.7% and a 90-day mortality of 4.9%, with postoperative complication rates ranging from 50 to 88% for low-grade and 30 to 42% for high-grade Clavien–Dindo complications, and is well recognised to significantly impact health-related quality of life (HRQoL) [8,9,10], consistent with more recent series [11,12].

The question of whether the timing of RC influences perioperative and patient-reported outcomes remains insufficiently studied, as existing comparative data have focused predominantly on oncological endpoints. A systematic review pooling ten retrospective series (n = 1516) reported a survival benefit with early cystectomy in high-grade NMIBC (hazard ratio for deferred versus early RC = 1.24; *p* = 0.029) [13]; RC also represents overtreatment for a significant minority and greatly impairs quality of life [8,9,10]. Whether prior BCG exposure modulates postoperative HRQOL trajectories following RC has not been systematically evaluated. The aim of the present study was therefore to compare perioperative outcomes and longitudinal HRQOL between patients undergoing early RC (without prior BCG) and delayed RC (following BCG therapy) for high-risk NMIBC at a tertiary referral centre.

## 2. Patients and Methods

### 2.1. Patient Selection

At our tertiary referral centre, we retrospectively analysed a prospectively maintained database. Patients that underwent radical cystectomy between April 2013 and November 2023 for EAU very-high-risk urothelial cell carcinoma and patients after BCG refractory disease according to current EAU guidelines were included [3]. All cases were retrospectively reclassified according to the single, contemporary EAU risk-stratification system to ensure uniform group definition. Patients with variant histology were excluded. Ethical approval has been obtained from the local ethical committee of LMU University (approval no.: 24-0650).

General clinical and surgery-related data were obtained from the underlying database. Information about previous intravesical therapy was obtained from previous letters and/or the treating physicians/urologists. Among the 45 delayed-RC patients, the number of preoperative BCG instillations was documented in 43 (median 6, IQR 1.5–6, range 1–24), of whom 27 (62.8%) completed a full 6-instillation induction course; 8 patients (17.8%) discontinued BCG because of treatment-related adverse events. Maintenance BCG status was documented in only 10 of 45 patients (9 of whom received at least one maintenance cycle), and instillation dose and adverse event grade were not systematically recorded in the chart, precluding a more granular analysis of BCG exposure.

Health-related quality of life questionnaires (EORTC QLQ-C30) are routinely given to the patients the day before surgery as well as 3 months, 12 months, and every year postoperative. Questionnaires were analysed using the EORTC scoring manual. Symptom scores range from 100 to 0, and a low score refers to a low symptom burden. Functioning scores range from 0 to 100, and a high score equals high functioning in the respective domain. The global health status (GHS) (questions 29 and 30 of the QLQ-C30 questionnaire) refers to the general quality of living, with higher scores accounting for a better HRQOL.

### 2.2. Statistics

Continuous data are presented as medians and interquartile ranges (IQRs), whereas categorical data are reported as counts and percentages. Normality of continuous variables was assessed using the Shapiro–Wilk test. Group comparisons were conducted using either the independent-samples *t*-test for normally distributed and the Mann–Whitney U test for non-normally distributed continuous variables, and the chi-square or Fisher’s exact test for categorical variables, as appropriate given expected cell counts. Longitudinal changes in all QLQ-C30 domains were assessed using linear mixed-effects models with patient-level random intercepts and a group-by-time interaction term, which accommodates all available repeated measures without requiring complete-case exclusion. Models were adjusted for age and preoperative CIS status; urinary diversion type was not included as a covariate given extensive missingness in the early-RC group (70/81 undocumented) that would otherwise have restricted several models to a small, unbalanced subsample. *p*-values for the group-by-time interaction were corrected for multiple comparisons across all domains and timepoints using the Benjamini–Hochberg false discovery rate (FDR) procedure. As sensitivity analyses, we additionally applied stabilised inverse probability of treatment weighting (age, CIS, tumour configuration, cardiovascular/pulmonary comorbidity, calendar year) and repeated the primary comparisons after excluding patients with CIS. Overall survival between the two groups was assessed using the Kaplan–Meier method. Statistical analyses were performed with R software (version 3.6.3, R Foundation for Statistical Computing, Vienna, Austria), and statistical significance was defined as a two-tailed *p* value < 0.05. As this was a retrospective analysis of a fixed, already-accrued cohort, no a priori sample size or power calculation was performed. To characterise the precision of the primary GHS comparison, we instead calculated the minimum detectable effect (MDE) at 80% power and a two-sided alpha of 0.05, given the observed sample sizes and pooled standard deviation at each timepoint (see Limitations).

## 3. Results

### 3.1. Baseline Characteristics

Overall, 126 patients who underwent RC were included in the analysis and stratified into two groups. Baseline characteristics are summarised in Table 1.

The two cohorts were well-balanced with respect to age, sex distribution (78% male in the early vs. 87% in the delayed group; *p* = 0.3), BMI, smoking history (61% vs. 60%; *p* > 0.9), and ECOG performance status (*p* = 0.4). The preoperative tumour stage differed significantly between groups (*p* < 0.001), with pT1 tumours being more prevalent in the early RC group (77% vs. 43%) and CIS more common in the delayed group (39% vs. 10%). As expected, the median time from initial diagnosis to cystectomy was substantially longer in the delayed group (56.9 weeks, IQR 27–111.4) compared with the early group (8.6 weeks, IQR 6–18.0; *p* < 0.001). Median overall follow-up was 27 months.

### 3.2. Perioperative and Histopathological Outcomes

Data are detailed in Table 2. Urinary diversion with orthotopic neobladder was performed in 68.9% of delayed RC patients vs. 4.9% of early RC patients (*p* = 0.080). Surgery duration (209.5 vs. 217.0 min; *p* = 0.8) and intraoperative blood loss (300 mL in both groups; *p* = 0.900) were comparable. The overall perioperative transfusion rate was 9.7%, with a non-significant trend toward lower rates in the delayed group (*p* = 0.080). ICU admission rates were significantly higher in the early RC group (37% vs. 18%; *p* = 0.040). The overall postoperative complication rate was 34% in the early and 36% in the delayed group with no significant difference in the rate of severe complications (Clavien-Dindo ≥ III: 44% vs. 41%; *p* > 0.900). Median length of hospital stay was the same in both groups.

Histopathological results revealed urothelial differentiation in 94% and 100% of early and delayed RC patients, respectively. A complete pathological response (pT0) was observed in 16% of early and 21% of delayed RC patients (*p* = 0.7). There was no significant difference in the presence of locally advanced disease, lymph node positivity, or positive resection margins (*p* > 0.05).

### 3.3. Health-Related Quality of Life (EORTC QLQ-C30)

Evaluable QLQ-C30 questionnaires were available for 126 patients (81 early, 45 delayed) at baseline, 93 (63/30) at 3 months, 89 (54/35) at 12 months, and 70 (45/25) at 24 months. Missing follow-up questionnaires were not imputed; the mixed-effects models described below use all available observations without requiring complete follow-up. Preoperative functioning scores were well-matched between groups across all five EORTC QLQ-C30 domains (all *p* ≥ 0.05; Supplementary Table S1). Physical functioning [9] declined from a preoperative median of 93.0 in both groups to 86.7 at 3 months, with partial recovery at 24 months (86.7 in the early and 90.0 in the delayed group), without significant between-group differences at any timepoint (all *p* ≥ 0.7). Role functioning (RF) showed a similar pattern as displayed in Figure 1A,B.

Within-group analyses revealed statistically significant changes over the 24-month observation period in functioning domains. In the early RC group, significant within-group improvements were observed for physical functioning (*p* = 0.000105), role functioning (*p* = 0.000937), social functioning (*p* = 0.0107), and emotional functioning (*p* = 0.0123). In the delayed RC group, significant within-group changes were observed for role functioning (*p* = 0.0165), cognitive functioning (*p* = 0.0122), and GHS (*p* = 0.0482), whereas physical functioning showed only a trend toward significance (*p* = 0.0517).

Preoperative symptom burden was low and comparable between groups across all assessed symptom scales (all *p* > 0.05; Supplementary Table S1). Postoperatively, fatigue was the most consistently reported symptom, with scores increasing from 22.2 preoperative to 33.3 at 3 months in both groups, before partially recovering to 22.2 and 33.3 at 24 months in the early and delayed groups, respectively (all between-group *p* ≥ 0.4). Insomnia scores increased from 0.0 preoperative to 33.3 at 3 months in both cohorts and remained elevated at 24 months, without significant between-group differences (all *p* ≥ 0.7). Symptom score changes from baseline are displayed in Figure 2.

### 3.4. General Quality of Life (Global Health Status)

Preoperative GHS scores were comparable between the early and delayed RC groups (median 66.7; *p* = 0.10), as shown in Figure 1C. At 3 months postoperative, GHS remained unchanged at 66.7 in both groups (*p* = 0.8). By 12 months, a modest improvement was observed in both cohorts (early: 70.8, IQR 50–83.3; delayed: 75.0, IQR 63–83.3; *p* = 0.4), with further stabilisation at 24 months (75.0 in both; *p* = 0.9). The proportion of patients with a GHS > 70 increased over time in both groups from 49% and 36% preoperative to 51% and 60% at 24 months in the early and delayed RC groups without showing a significant difference between early and delayed RC. Changes of functioning scores and GHS from baseline to 24 months postoperative are displayed in Figure 3.

In linear mixed-effects models adjusted for age and CIS status, the group-by-time interaction for GHS was not statistically significant at 3 months (Coefficient = 2.2; *p* = 0.678) or 24 months (Coefficient = 6.1; *p* = 0.295). At 12 months, a nominal difference favouring the delayed RC group was observed (Coefficient = 10.5, 95% CI 0.2–20.9; *p* = 0.046), but this did not remain significant after FDR correction for multiple comparisons across all QLQ-C30 domains and timepoints (p_FDR = 0.776). No domain-by-timepoint comparison retained significance after correction.

### 3.5. Survival Analysis

Overall survival did not differ significantly between the two groups over a follow-up of up to 60 months, as shown in Figure 1. Kaplan–Meier analysis revealed comparable survival curves for early and delayed RC patients (*p* = 0.720).

To account for confounding by indication, we performed stabilised inverse probability of treatment weighting (IPTW). A propensity score model (logistic regression) incorporated age, preoperative CIS, solid tumour configuration, cardiovascular disease, pulmonary disease, and calendar year of surgery. Tumour size was excluded given 82% missingness, and urinary diversion type was excluded because it is a postoperative, treatment-related variable rather than a baseline confounder and was itself extensively missing in the early-RC group (70/81 undocumented). Weighting achieved good covariate balance across all included variables (Table 3; standardised mean differences <0.16 after weighting, versus up to 0.46 unweighted). Under IPTW, GHS did not differ significantly between groups at any timepoint (all *p* ≥ 0.30), and overall survival remained non-significant in a weighted Cox model (HR 1.71, 95% CI 0.76–3.86; *p* = 0.20), consistent with the primary analysis.

Cancer-specific survival (CSS) events were rare across the cohort (2/126 patients: 1/81 early RC, 1/45 delayed RC); unadjusted CSS at last follow-up was 97.9% in the early RC group versus 97.7% in the delayed RC group (log-rank *p* = 0.724). Given only two cancer-specific deaths, a multivariable or weighted CSS analysis was not performed, as it would not yield a reliable estimate. Recurrence-free and progression-free survival could not be assessed, as post-cystectomy recurrence and progression events were not systematically captured as structured, dated variables in this retrospective single-centre database.

## 4. Discussion

In our retrospective cohort study of 126 patients with very-high-risk NMIBC undergoing radical cystectomy, we observed no significant differences in perioperative outcomes or longitudinal HRQOL between patients who underwent early RC without prior BCG and those who proceeded to delayed RC following prior BCG therapy. Survival outcomes were comparable between groups.

Regarding perioperative outcomes, the two groups were largely comparable in terms of surgery duration, intraoperative blood loss, overall complication rates, severity of complications (Clavien-Dindo ≥ III), and length of hospital stay. These findings are consistent with existing literature: prior multicentre analyses have reported similar perioperative outcomes between upfront and delayed early RC for HR-NMIBC [14]. Diamant et al. demonstrated that there were no significant differences between early and delayed radical cystectomy with respect to operative time (315 vs. 300 min; *p* = 0.948), estimated blood loss (800 vs. 700 mL; *p* = 0.498), or perioperative blood transfusion rates. The one significant perioperative difference observed in our cohort was a higher ICU admission rate in the early RC group (37% vs. 18%; *p* = 0.040). Advanced age and comorbidity burden are known independent risk factors for ICU admission following radical cystectomy [15], so we tested this directly in a multivariable logistic regression model adjusting for age, ASA class, and cardiovascular, pulmonary, and metabolic comorbidity. The association between treatment group and ICU admission persisted after adjustment (early versus delayed RC: adjusted OR 2.74, 95% CI 1.05–7.18; *p* = 0.040), independent of age (adjusted OR 1.08 per year, 95% CI 1.02–1.14; *p* = 0.005); no individual comorbidity was independently associated with ICU admission. This indicates that the higher ICU admission rate in the early RC group is not fully explained by the baseline age and comorbidity data captured in our database and more likely reflects institutional triage practice or unmeasured selection factors. Importantly, this difference in ICU admission did not translate into higher overall complication rates or a prolonged hospital stay, suggesting that it reflects an institutional triage practice rather than inferior perioperative safety. The longer interval to cystectomy in the delayed group reflects, by definition, the time required to complete a trial of BCG induction (and, in most patients, at least one maintenance cycle) before treatment failure was declared, rather than a diagnostic or logistical delay. Similarly, the higher proportion of orthotopic neobladder in the delayed group likely reflects a combination of factors: patients who remained candidates for BCG were, by definition, fit for observation and eventual surgery, and the more localised disease pattern in this group (more CIS, less pT1) at the time of cystectomy may have made continent diversion oncologically appropriate more often, in addition to evolving centre practice over the study period; a watch-and-wait approach to diversion choice cannot be excluded but was not systematically documented. To further address confounding by indication, we performed two sensitivity analyses. First, we constructed stabilised inverse probability of treatment weights (IPTW); the covariate balance achieved and the resulting effect estimates are reported in the Results (Table 3). As with any propensity-based method, IPTW can only balance the observed covariates included in the model; unmeasured confounders, such as comorbidity severity, functional status, or unrecorded treatment preference, may still bias the comparison, and the concordance of the weighted estimates with the primary analysis increases confidence without eliminating this possibility. Second, restricting the cohort to patients without CIS (n = 101: 73 early, 28 delayed) to reduce heterogeneity, as suggested, showed no significant between-group differences in overall complications or GHS at any timepoint, and a numerically lower ICU admission rate in the delayed group that did not reach significance (18% vs. 40%; *p* = 0.059) (Supplementary Table S2), consistent with the primary analysis.

The pathological complete response rate (pT0) was numerically higher in the delayed RC group (21% vs. 16%), albeit without reaching statistical significance (*p* = 0.7). This is broadly consistent with contemporary series reporting pT0 rates and their predictors after radical cystectomy [16], although the effect of prior BCG exposure on pT0 achievement remains incompletely understood; given the small sample size and the non-significant result, this finding should be interpreted with caution. Lymph node positivity and positive resection margins were rare and comparable between groups, further supporting the oncological equivalence of both treatment strategies in the perioperative setting.

Contrary to our initial analysis based on multivariable linear regression, longitudinal mixed-effects modelling with patient-level random intercepts and a group-by-time interaction—which uses all available repeated measures rather than only complete cases—did not identify a robust between-group difference in GHS or in any other QLQ-C30 domain at any postoperative timepoint. A nominal difference in GHS favouring the delayed RC group at 12 months (Coefficient = 10.5; unadjusted *p* = 0.046) did not survive correction for multiple comparisons across all domains and timepoints (FDR-adjusted *p* = 0.776) and should not be interpreted as a genuine treatment-timing effect. We note that our earlier complete-case regression model, which additionally adjusted for urinary diversion type, was based on markedly smaller and unbalanced subsamples at several timepoints (as few as 7 early-RC patients at 12 months and 1 at 24 months) because urinary diversion status was undocumented in the majority (70/81) of early-RC patients; this likely explains the isolated large effect estimate previously reported. The consistent absence of a between-group difference across the full HRQOL profile, rather than an isolated transient effect, provides more robust support for the conclusion that the timing of RC relative to BCG therapy does not meaningfully alter postoperative quality of life. The higher proportion of orthotopic neobladder use in the delayed RC group (68.9% vs. 4.9%) remains a relevant descriptive difference between groups; however, its association with HRQOL could not be reliably assessed given the extensive missingness in urinary diversion documentation in the early-RC group and is addressed further under Limitations.

In line with previous literature, overall survival did not differ significantly between the two groups over a follow-up of up to 60 months (*p* = 0.72). A contemporary cohort study comparing BCG and early RC in patients with very-high-risk NMIBC similarly found no differences in overall survival between treatment strategies (OS rates 80,2% in the BCG and 88,1% non-BCG group, *p* = 0.600) [17]. In another large European multicentre cohort, patients treated with BCG did not exhibit a cancer-specific mortality disadvantage relative to those undergoing upfront RC, with delayed RC matching upfront RC outcomes when performed before disease progression [18]. The absence of a survival difference in our cohort underlines that, in carefully selected patients managed at a tertiary centre with structured follow-up, a trial of BCG therapy does not compromise long-term oncological outcomes.

Yet, the current study has several limitations that must be acknowledged. The retrospective, single-centre design limits generalisability and introduces inherent selection bias, as the allocation to early versus delayed RC was not randomised but determined by clinical factors and patient preference. The two groups differed significantly in preoperative tumour stage distribution and the proportion of patients with CIS, reflecting the real-world heterogeneity of very-high-risk NMIBC and the evolving, risk-adapted management of CIS within contemporary NMIBC care [19]. These differences, together with the markedly longer time to cystectomy in the delayed group, are not random variation but a direct consequence of the clinical indication for BCG versus upfront surgery, and confounding by indication cannot be fully resolved even with the sensitivity analyses described above. Although key confounders were accounted for in the multivariable models, residual confounding cannot be excluded despite the concordant IPTW-weighted estimates. Oncological endpoints beyond overall survival were similarly constrained by the retrospective, single-centre design: only two cancer-specific deaths occurred during follow-up, precluding a robust cancer-specific survival analysis, and recurrence-free and progression-free survival could not be assessed at all because post-cystectomy recurrence and progression events were not systematically captured as structured, dated variables in the underlying database. Overall survival should therefore be interpreted as an imprecise proxy for oncological equivalence between the two strategies. As the sample size was fixed by the retrospective cohort rather than derived from an a priori power calculation, the risk of a type II error must be considered when interpreting the non-significant GHS comparisons: at 80% power and a two-sided alpha of 0.05, the study could detect a minimum between-group difference of approximately 12 points in GHS at baseline and 3 months, and approximately 13–15 points at 12 and 24 months, exceeding the established minimally important difference of 10 points for the QLQ-C30 [20]. A true difference smaller than this, including one at or near the clinically important threshold, could therefore have gone undetected, and the absence of a significant difference should not be interpreted as evidence of equivalence. Finally, the EORTC QLQ-C30, while well-validated as a general oncological instrument, does not capture bladder cancer-specific QOL domains such as urinary function or stoma-related outcomes, which are of particular relevance following RC and urinary diversion. Data on prior BCG therapy were similarly limited by the retrospective, chart-based ascertainment: while the number of induction instillations and discontinuation due to adverse events were well documented, maintenance BCG status was recorded in only a minority of patients, and instillation dose and adverse event severity were not systematically captured, so that distinct scenarios of BCG exposure and failure could not be further differentiated within the delayed-RC group.

Finally, we were able to show that delayed radical cystectomy following prior BCG therapy does not adversely affect survival outcomes, and importantly, our study is the first to demonstrate that HRQOL does not differ between the two groups.

## 5. Conclusions

In conclusion, perioperative outcomes or longitudinal HRQOL following radical cystectomy for very-high-risk NMIBC was not adversely influenced by prior BCG therapy, with QOL trajectories and overall survival comparable between groups at 24 months. These findings support the initial use of BCG in eligible patients, as a trial of intravesical therapy does not appear to compromise surgical safety or postoperative quality of life when cystectomy is ultimately required. Finally, prospective randomised trials are warranted to further substantiate the evidence generated by our study.

## Figures and Tables

**Figure 1:**
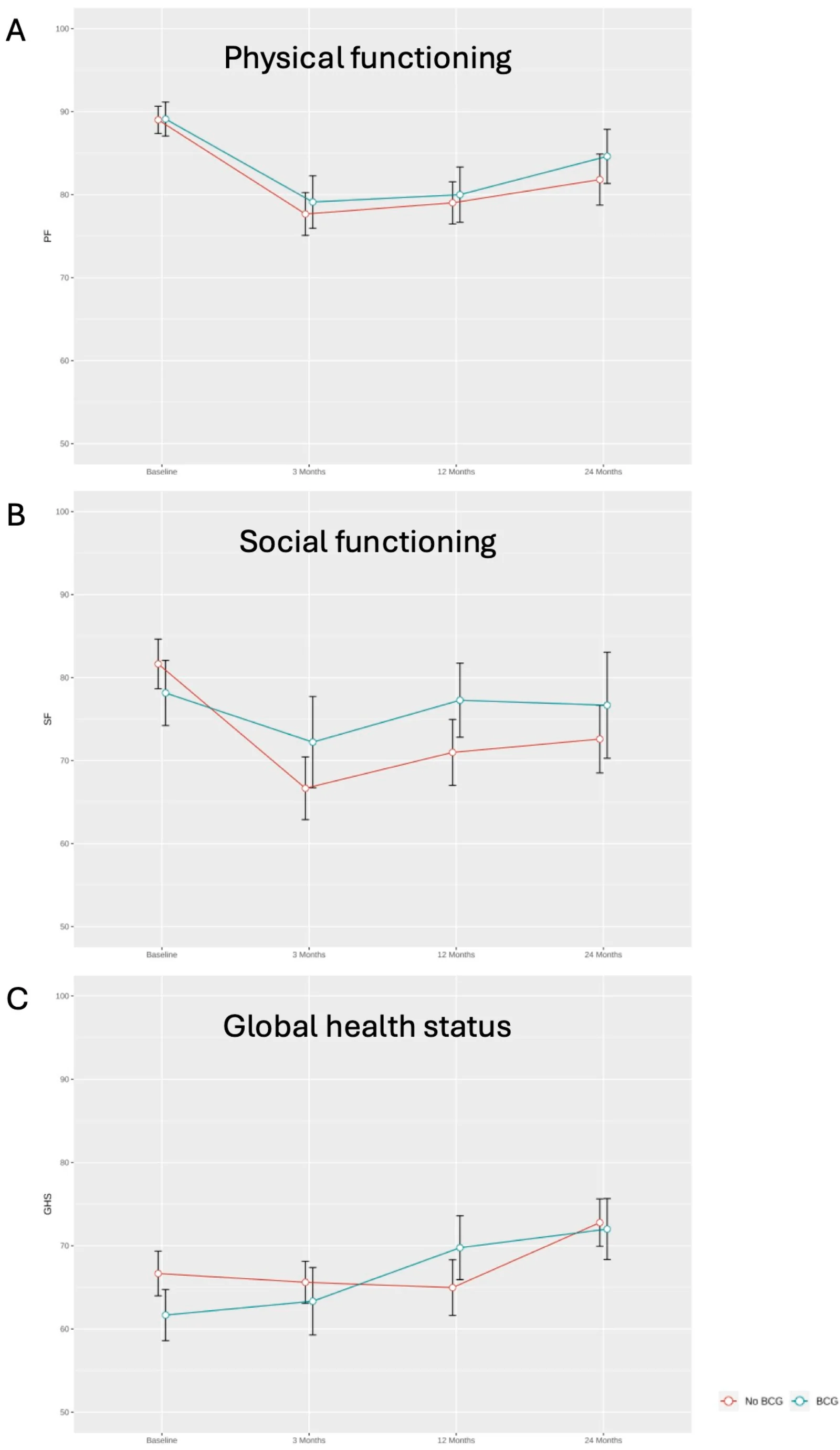
Longitudinal changes in patients with early and delayed cystectomy in (**A**) physical functioning subscale, (**B**) social functioning subscale, and (**C**) global health status.

**Figure 2:**
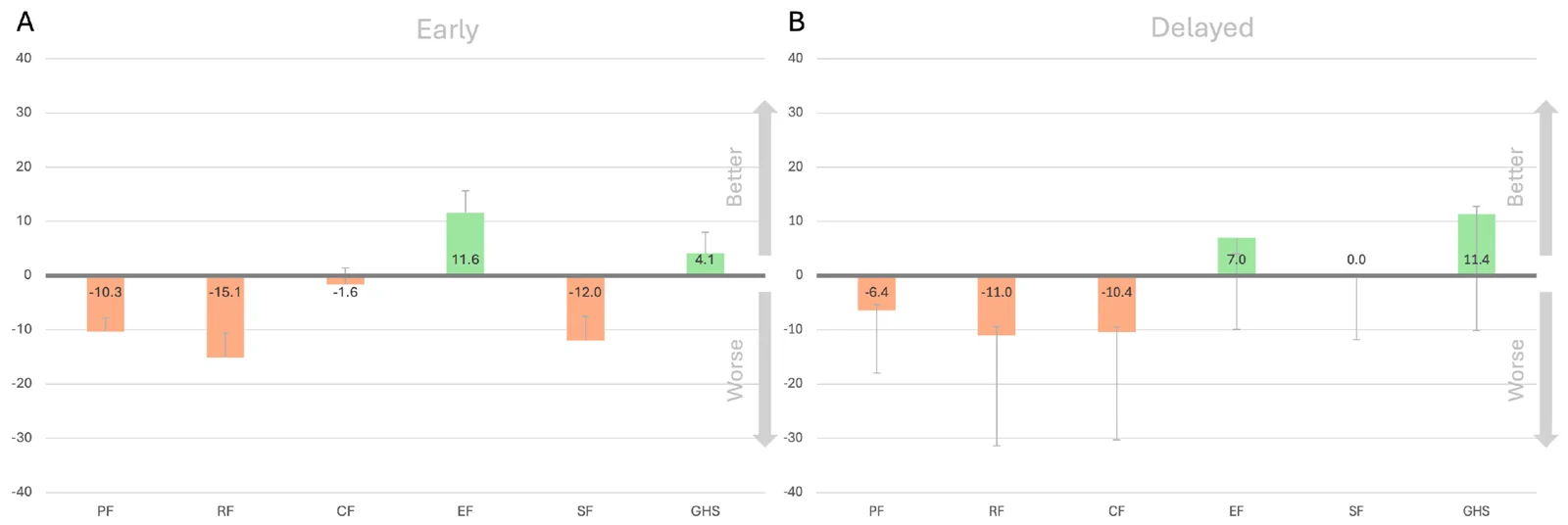
Changes in EORTC symptom scores from baseline to 24 months postoperative in patients with (**A**) early and (**B**) delayed cystectomy.

**Figure 3:**
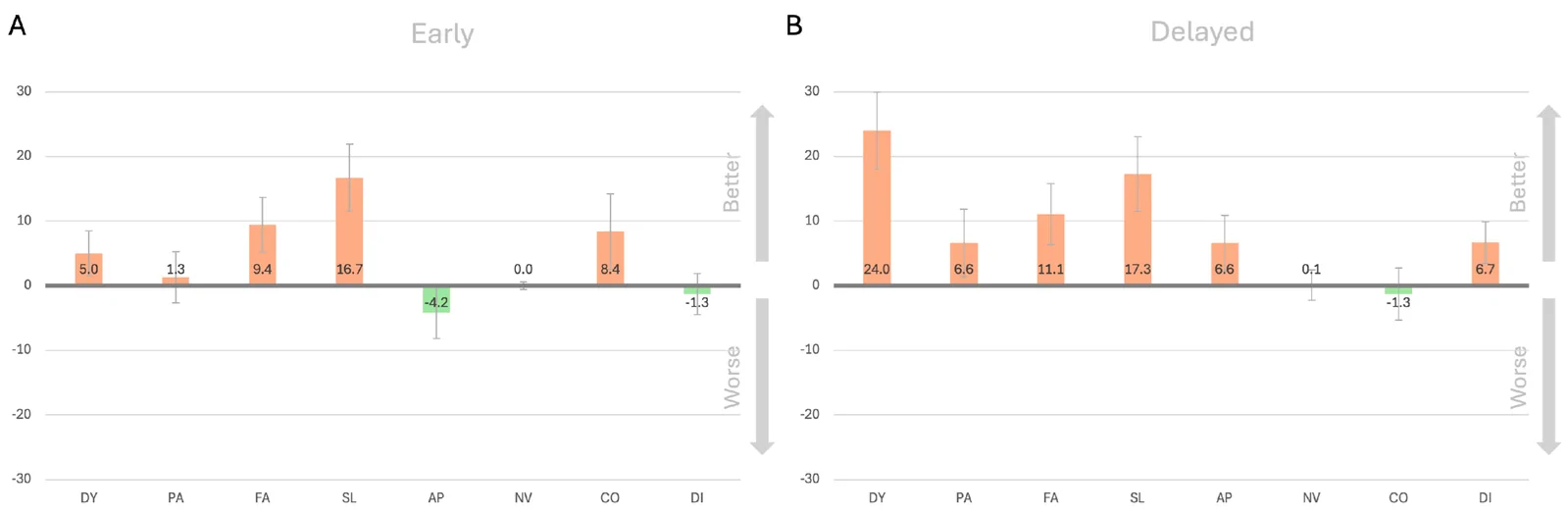
Changes in EORTC functioning scores from baseline to 24 months postoperative in patients with (**A**) early and (**B**) delayed cystectomy.

**Table 1:** Baseline characteristics in patients with early and delayed cystectomy after BCG.

Characteristic	Overall, n = 126	Early (n = 81)	Delayed (n = 45)	*p*-Value
Sex				0.3
Female n, (%)	24 (19%)	18 (22%)	6 (13%)	
Male n, (%)	102 (81%)	63 (78%)	39 (87%)	
Age, median (IQR)	69.6 (63–76.2)	71.0 (63–78.0)	69.0 (62–75.0)	0.2
ASA ≥ 3	95 (75%)	58 (72%)	37 (82%)	0.3
Histopathological result preoperative TUR-BT, n (%)				**<0.001**
pTa	18 (15%)	10 (13%)	8 (18%)	
pT1	79 (65%)	60 (77%)	19 (43%)	
CIS	25 (20%)	8 (10%)	17 (39%)	
Grading TUR-BT preoperatively (high grade), n (%)	122 (97%)	78 (96%)	44 (98%)	>0.9
Solid tumour configuration	13 (10%)	12 (15%)	1 (2.2%)	0.055
Tumour size	3.7 (2–9.0)	4.3 (3–10.2)	2.7 (2–6.9)	0.052
Time to cystectomy, weeks	14.6 (7–50.1)	8.6 (6–18.0)	56.9 (27–111.4)	**<0.001**
BMI	26.2 (24–29.9)	25.8 (24–29.7)	26.4 (25–31.0)	0.2
Smoking history	73 (61%)	49 (61%)	24 (60%)	>0.9
Cardiovascular comorbidity	75 (60%)	47 (58%)	28 (62%)	0.8
Pulmonary comorbidity	24 (19%)	14 (17%)	10 (22%)	0.7
Endocrinological disorder	45 (36%)	31 (38%)	14 (31%)	0.5
ECOG				0.4
0	93 (76%)	60 (76%)	33 (75%)	
1	26 (21%)	15 (19%)	11 (25%)	
2	2 (1.6%)	2 (2.5%)	0 (0%)	
3	2 (1.6%)	2 (2.5%)	0 (0%)	

Tumour size was documented for 22/126 patients (16 early, 6 delayed) and is reported as the median (IQR) of available data only; solid tumour configurations were documented for all 126 patients. Bold values indicate statistical significance.

**Table 2:** Perioperative and surgical outcomes.

Characteristic	Overall, n = 126	Early (n = 81)	Delayed (n = 45)	*p*-Value
Urinary diversion with neobladder	35 (27.8%)	4 (4.9%)	31 (68.9%)	0.080
Surgery duration	214.0 (185–254.0)	209.5 (186–260.0)	217.0 (185–240.0)	0.8
Blood loss	300.0 (200–600.0)	300.0 (100–600.0)	300.0 (200–500.0)	0.9
Transfusion rate	12 (9.7%)	11 (14%)	1 (2.3%)	0.080
ICU admission	38 (30%)	30 (37%)	8 (18%)	0.040
Any postoperative complication	43 (34%)	27 (34%)	16 (36%)	>0.9
Clavien ≥ 3	19 (43%)	12 (44%)	7 (41%)	>0.9
Lenght of hospital stay	19.0 (16–22.0)	19.0 (15–22.0)	19.0 (16–22.0)	0.3
Histopathological results				
Urothelial differentiation	121 (96%)	76 (94%)	45 (100%)	0.2
pT0	21 (18%)	12 (16%)	9 (21%)	0.7
Locally advanced	10 (8.6%)	8 (11%)	2 (4.7%)	0.4
Lymph node status	9 (7.1%)	6 (7.4%)	3 (6.7%)	>0.9
Resection status	2 (1.6%)	2 (2.5%)	0 (0%)	0.7

**Table 3:** Covariate balance before and after inverse probability of treatment weighting (IPTW).

Covariate	SMD, Unweighted	SMD, Weighted
Age	−0.17	−0.01
Preoperative CIS	0.35	−0.08
Solid tumour configuration	−0.46	0.03
Cardiovascular disease	0.09	−0.05
Pulmonary disease	0.12	0.01
Calendar year of surgery	−0.30	−0.16

## Data Availability

The data presented in this study are available on request from the corresponding author due to ethical reasons.

## Supplementary Materials

The following supporting information can be downloaded at: https://www.mdpi.com/article/10.3390/jcm15197486/s1, Table S1: EORTC subscales at baseline and 3, 12 and 24 months postoperatively; Table S2. Sensitivity analysis excluding patients with carcinoma in situ (CIS).
