# Supplementary material for: Does Timing Matter? Perioperative Outcomes and Quality of Life After Early Versus Delayed Radical Cystectomy Following BCG Therapy
Source: J Clin Med. 2026 Sep 26;15(19):7486. doi: 10.3390/jcm15197486 (PMC13666204; doi:10.3390/jcm15197486)
Supplement: Supplementary file 1 [file jcm-15-07486-s001.zip › jcm-4539960-supplementary.pdf]

Supplementary Table S1 - EORTC subscales at baseline and 3, 12 and 24 months postoperatively

**EORTC scale**	**Overall** (n=126)	**Early** (n= 81)	**Delayed** (n= 45)	**p-value**[2]
		**Functioning domains**		
Physical functioning preop	93.0 (80-100.0)	93.0 (80-100.0)	93.0 (80-100.0)	0.7
Physical functioning 3 months	86.7 (67-93.3)	86.7 (63-93.3)	86.7 (67-93.3)	>0.9
Physical functioning 12 months	86.7 (67-93.3)	83.3 (67-93.3)	86.7 (70-93.3)	0.8
Physical functioning 24 months	86.7 (73-100.0)	86.7 (67-100.0)	90.0 (73-100.0)	0.7
Role functioning preop	100.0 (67-100.0)	100.0 (67-100.0)	100.0 (67-100.0)	0.8
Role functioning 3 months	66.7 (50-83.3)	66.7 (42-83.3)	66.7 (50-66.7)	0.9
Role functioning 12 months	66.7 (50-100.0)	66.7 (33-100.0)	66.7 (67-100.0)	0.14
Role functioning 24 months	83.3 (50-100.0)	83.3 (50-100.0)	83.3 (67-100.0)	0.5
Cognitive functioning preop	100.0 (83-100.0)	100.0 (83-100.0)	100.0 (100-100.0)	0.054
Cognitive functioning 3 months	100.0 (83-100.0)	100.0 (83-100.0)	100.0 (83-100.0)	0.4
Cognitive functioning 12 months	100.0 (79-100.0)	100.0 (67-100.0)	100.0 (83-100.0)	0.12
Cognitive functioning 24 months	91.7 (83-100.0)	100.0 (83-100.0)	83.3 (83-100.0)	0.7
Emotional functioning preop	75.0 (58-91.7)	75.0 (56-91.7)	75.0 (58-83.3)	0.7
Emotional functioning 3 months	83.3 (58-100.0)	83.3 (58-100.0)	75.0 (60-91.7)	0.7
Emotional functioning 12 months	83.3 (67-100.0)	83.3 (52-100.0)	83.3 (67-100.0)	0.5
Emotional functioning 24 months	83.3 (67-100.0)	91.7 (65-100.0)	83.3 (67-91.7)	0.5
Social functioning preop	100.0 (67-100.0)	100.0 (67-100.0)	83.3 (67-100.0)	0.4
Social functioning 3 months	66.7 (50-100.0)	66.7 (50-100.0)	83.3 (50-100.0)	0.4
Social functioning 12 months	66.7 (67-100.0)	66.7 (50-100.0)	83.3 (67-100.0)	0.4
Social functioning 24 months	83.3 (54-100.0)	66.7 (50-100.0)	100.0 (67-100.0)	0.4
Global health status preop	66.7 (50-83.3)	66.7 (58-83.3)	66.7 (50-75.0)	0.10
Global health status 3 months	66.7 (50-83.3)	66.7 (50-83.3)	66.7 (50-83.3)	0.8
Global health status 12 months	75.0 (50-83.3)	70.8 (50-83.3)	75.0 (63-83.3)	0.4
Global health status 24 months	75.0 (58-83.3)	75.0 (58-83.3)	75.0 (67-83.3)	0.9
		**Symptom domains**		
Dyspnoe preop	0.0 (0-33.3)	0.0 (0-33.3)	0.0 (0-0.0)	0.067
Dyspnoe 3 months	16.7 (0-33.3)	33.3 (0-33.3)	0.0 (0-33.3)	0.8
Dyspnoe 12 months	0.0 (0-33.3)	0.0 (0-33.3)	0.0 (0-58.3)	0.8

EORTC scale	Overall (n=126)	Early (n= 81)	Delayed (n= 45)	p-value[2]
Dyspnoe 24 months	0.0 (0-33.3)	0.0 (0-33.3)	16.7 (0-33.3)	0.5
Pain preop	0.0 (0-16.7)	0.0 (0-16.7)	0.0 (0-33.3)	0.6
Pain 3 months	0.0 (0-33.3)	0.0 (0-33.3)	16.7 (0-33.3)	0.3
Pain 12 months	0.0 (0-33.3)	0.0 (0-33.3)	0.0 (0-16.7)	0.3
Pain 24 months	0.0 (0-33.3)	0.0 (0-33.3)	0.0 (0-33.3)	0.6
Fatique preop	22.2 (11-33.3)	22.2 (0-33.3)	11.1 (11-33.3)	0.5
Fatique 3 months	33.3 (22-55.6)	33.3 (22-50.0)	33.3 (22-55.6)	0.7
Fatique 12 months	33.3 (11-55.6)	33.3 (11-55.6)	33.3 (11-44.4)	0.4
Fatique 24 months	27.8 (0-44.4)	22.2 (0-55.6)	33.3 (14-41.7)	0.9
Insomnia preop	0.0 (0-33.3)	0.0 (0-33.3)	0.0 (0-33.3)	>0.9
Insomnia 3 months	33.3 (33-66.7)	33.3 (0-66.7)	33.3 (33-66.7)	0.7
Insomnia 12 months	33.3 (33-66.7)	33.3 (33-66.7)	33.3 (17-66.7)	0.8
Insomnia 24 months	33.3 (0-66.7)	33.3 (0-66.7)	33.3 (0-58.3)	0.8
Appetite loss preop	0.0 (0-0.0)	0.0 (0-0.0)	0.0 (0-0.0)	0.2
Appetite loss 3 months	0.0 (0-0.0)	0.0 (0-16.7)	0.0 (0-0.0)	0.8
Appetite loss 12 months	0.0 (0-0.0)	0.0 (0-0.0)	0.0 (0-0.0)	0.6
Appetite loss 24 months	0.0 (0-0.0)	0.0 (0-0.0)	0.0 (0-0.0)	0.5
Nausea/vomiting preop	0.0 (0-0.0)	0.0 (0-0.0)	0.0 (0-0.0)	0.6
Nausea/vomiting 3 months	0.0 (0-0.0)	0.0 (0-0.0)	0.0 (0-0.0)	0.5
Nausea/vomiting 12 months	0.0 (0-0.0)	0.0 (0-0.0)	0.0 (0-0.0)	>0.9
Nausea/vomiting 24 months	0.0 (0-0.0)	0.0 (0-0.0)	0.0 (0-0.0)	0.4
Constipation preop	0.0 (0-0.0)	0.0 (0-0.0)	0.0 (0-0.0)	0.8
Constipation 3 months	0.0 (0-33.3)	0.0 (0-33.3)	0.0 (0-33.3)	0.8
Constipation 12 months	0.0 (0-33.3)	0.0 (0-33.3)	0.0 (0-33.3)	0.5
Constipation 24 months	0.0 (0-33.3)	0.0 (0-33.3)	0.0 (0-33.3)	0.9
Diarrhoea preop	0.0 (0-0.0)	0.0 (0-0.0)	0.0 (0-0.0)	0.5
Diarrhoea 3 months	0.0 (0-33.3)	0.0 (0-33.3)	0.0 (0-0.0)	0.2
Diarrhoea 12 months	0.0 (0-33.3)	0.0 (0-33.3)	0.0 (0-25.0)	0.5
Diarrhoea 24 months	0.0 (0-33.3)	0.0 (0-33.3)	33.3 (0-33.3)	0.2
Financial instability preop	0.0 (0-0.0)	0.0 (0-0.0)	0.0 (0-8.3)	0.14

**EORTC scale**	**Overall** (n=126)	**Early** (n= 81)	**Delayed** (n= 45)	**p-value**[2]
Financial instability 3 months	0.0 (0-33.3)	0.0 (0-33.3)	0.0 (0-33.3)	0.4
Financial instability 12 months	0.0 (0-0.0)	0.0 (0-0.0)	0.0 (0-0.0)	0.9
Financial instability 24 months	0.0 (0-0.0)	0.0 (0-0.0)	0.0 (0-8.3)	0.8
**Good general global health status**				
GHS>70 preop	56 (44%)	40 (49%)	16 (36%)	0.2
GHS>70 3 months	43 (46%)	30 (48%)	13 (43%)	0.9
GHS>70 12 months	47 (53%)	27 (50%)	20 (57%)	0.7
GHS>70 24 months	38 (54%)	23 (51%)	15 (60%)	0.6

Supplementary Table S2 - Sensitivity analysis excluding patients with carcinoma in situ (CIS)

**Characteristic**	**Early (n = 73)**	**Delayed (n = 28)**	**p-value**
ICU admission, n (%)	29 (40%)	5 (18%)	0.059
Any postoperative complication, n (%)	25 (34%)	9 (32%)	>0.9
Age, median (IQR)	72.0 (63.0–78.0)	69.1 (63.8–75.0)	0.311
Surgery duration, min, median (IQR)	209.5 (187.5–260.0)	216.5 (192.5–247.0)	0.933
Length of hospital stay, days, median (IQR)	19.0 (15.0–22.0)	18.5 (16.0–21.0)	0.897
GHS, mixed-model coefficient (delayed vs. early), 3 months		1.8	0.786
GHS, mixed-model coefficient (delayed vs. early), 12 months		6.1	0.340
GHS, mixed-model coefficient (delayed vs. early), 24 months		5.4	0.452

Patients with CIS on preoperative TUR-BT were excluded (25/126: 8 early, 17 delayed), leaving 101 patients (73 early, 28 delayed). GHS coefficients are from the same linear mixed-effects model as the primary analysis (adjusted for age), restricted to this subgroup. Results are consistent with the primary analysis (no significant between-group differences), including a numerically lower ICU admission rate in the delayed group that did not reach significance (p=0.059) once CIS-related heterogeneity was removed.
